# Cumulative lifetime violence severity scale: development and initial testing among men

**DOI:** 10.1186/s12889-020-08551-6

**Published:** 2020-03-30

**Authors:** Kelly Scott-Storey, Sue O’Donnell, Judith Wuest, Judith MacIntosh, Marilyn Merritt-Gray

**Affiliations:** University of New Brunswick, Faculty of Nursing, P.O. Box 4400, Fredericton, New Brunswick E3B 5A3 Canada

**Keywords:** Cumulative lifetime violence severity, Scale development, Men, Health

## Abstract

**Background:**

Knowledge of the relationship between men’s health and violence is flawed by narrow and faulty conceptualization and measurement of violence that often results in attribution of health problems to one form or type of violence without consideration of other exposures. Our purpose is to describe the development and initial testing of the *Cumulative Lifetime Violence Severity* scale designed for use in health research to measure men’s perceptions of the severity of their cumulative lifetime violence.

**Methods:**

We framed the dimensions of violence severity as: type (physical, psychological, sexual), timing (childhood, adulthood), focus (perpetrator, target), context, frequency, and degree of distress. Items reflecting these dimensions were vetted by local experts including individuals who identified as men, with particular attention to meaningful language for men. The measure was pretested, revised to 64 items, and tested for test-retest reliability prior to use in a study of 685 English-speaking Eastern Canadian men, ages 19 to 65 years. We used Principal Components Analysis to illuminate the underlying dimensionality of the items.

**Results:**

Principal Components Analysis yielded a 44-item 11 component solution that accounted for 64.06% of variance with good model fit and a Cronbach’s alpha of .92. All dimensions of our conceptualization of violence severity were reflected in the components, except Adult Target Sexual Violence. Convergent validity between the Cumulative Lifetime Violence Severity-44 Scale and a global lifetime violence rating scale was *r =* .750 (*p* < .001) and concurrent validity was moderate and significant between the Cumulative Lifetime Violence Severity-44 scale and measures of mental health problems commonly experienced by people with violence histories.

**Conclusions:**

The Cumulative Lifetime Violence Severity-44 scale shows promise as the first comprehensive measure of cumulative lifetime violence for health research that considers gender, individual distress and experiences as both perpetrator and target. Next steps include further exploratory analysis with a more diverse sample of men and confirmatory factor analysis.

## Cumulative lifetime violence severity scale: development and initial testing among men

Despite accumulating evidence that violence is adversely related to health, knowledge of this complex relationship is flawed by narrow conceptualization and measurement of violence in health research [1]. There is urgent need to consider how health is affected by the accumulation of co-occurring and interconnecting multiple experiences of violence across the lifespan as perpetrator and target [2–4]. Yet measurement primarily relies on presence/absence or frequency of one or two types (i.e., physical, psychological, sexual) or contexts (intimate partner relationships, workplaces, families) at a particular point in time (e.g., childhood, adulthood) and ignores other dimensions of violence severity [5, 6]. We did not find any comprehensive measure of cumulative lifetime violence severity as target and perpetrator for use in studies of violence and men’s health. Our purpose is to describe the development and initial testing of the *Cumulative Lifetime Violence Severity* (CLVS) scale, designed for use in health research to measure men’s perceptions of the severity of their cumulative lifetime violence experience. We explain its conceptual underpinnings, describe its initial pilot testing and revision, and discuss examination of its factor structure and reliability in a community sample of 685 Eastern Canadian men.

## Background

### Conceptual underpinnings of cumulative lifetime violence severity

Men’s exposure to interpersonal violence within families, social networks, workplaces, and public spaces, both as perpetrators and targets, is pervasive throughout their lifetimes and has been shown to have negative effects on their health and well-being [7]. The term ‘target’ refers to someone who is the object of violence. Based on a critical analysis of studies of women’s health and violence, Scott-Storey concluded that negative health effects can rarely be attributed to one type or context of violence occurring during one period of the life course (i.e., childhood or adulthood) because multiple experiences of violence across the lifespan are common [1]. Although the body of research regarding men’s health and violence is less robust than that for women, these observations also apply to men. The full burden of violence can be understood only by considering the co-occurrence and interconnections among multiple, diverse, gendered experiences of violence including polyvictimization, polyperpetration, and perpetration-victimization [2]. Another limitation to current measurement of men’s violence exposure is emphasis on experiences as target and neglect of experiences as perpetrator.

Childhood exposure to violence in the home is a well-supported etiological factor for both victimization and perpetration. For example, early findings from the United States Adverse Childhood Events (ACE) study showed that the likelihood of intimate partner violence (IPV) perpetration by men and IPV victimization for women increased according to the cumulative number of types of childhood events (physical, sexual, witnessing) experienced [8]. More recently, Voith et al. (2017) extended this work, examining how childhood exposure to specific types (physical, sexual, and emotional as well as polyvictimization) of violence were related to *adult patterns* of perpetration and victimization among 423 college men; 27% reported polyperpetration, 43.5% reported polyvictimization, and 60% reported both victimization and perpetration in the previous year [4]. For adulthood, childhood physical abuse predicted both psychological and polyvictimization and perpetration as well as sexual victimization; childhood sexual abuse predicted physical and sexual perpetration and victimization as well as polyperpetration; and emotional abuse predicted physical and psychological victimization. These complex interconnections among violence experiences corroborate the need for a measure that captures the multiplicity and cooccurrence of violence experiences and the inclusion of experiences as both perpetrator and target. Despite evidence that the same etiological pathways (e.g., biological stress response, poverty) underpin models of perpetration and victimization, studies focusing on the intersections of these experiences are scarce [2]. Moreover, a strong reciprocal link between being both a target and perpetrator of interpersonal violence has been established within the criminology field [9].

Critical underpinnings of the CLVS scale are: a) men have multiple, diverse, and interconnected experiences of violence across their lifespans both as target and perpetrator, and b) the relationship between violence exposure and health must be understood within the context of the lifetime accumulation of these experiences.

#### Health

Experiences of violence can be traumatic events that evoke an adaptive acute stress response [10] that protects vital body functions through a process of allostasis [11]. However, recurrent intense stress such as that from violence experiences can produce allostatic overload that leads to dysregulation of the body’s natural stress response system, causing significant and persistent, biophysical (e.g., neuroendocrine, metabolic, immunologic) changes to the body and brain that have been implicated in the development and progression of many chronic diseases [12, 13]. An essential mechanism for precipitating changes in health and well-being in response to exposure to violence and other trauma is a *distress* reaction; that is, how distressing the experience is perceived to be [14]. Therefore, a key dimension of violence severity in health research is the degree of distress experienced from exposure to violence. Some evidence shows that how men conceptualize violence and how distressed they are consequent to being targets and/or perpetrators of violence differs from women [15]. Violence exposure, particularly IPV, for men often is measured using tools that were originally developed for women [16] and ignores gender, the lens through which violence is perceived, evaluated, expressed, and experienced [17, 18]. Thus, other critical underpinnings of the CLVS scale are gender and distress.

#### Severity

Researchers in violence and health often rely on a “more is worse” concept; that is, the dose-response relationship between health outcomes and the frequency of abuse exposure and/or the number of types of abuse, a practice that implies homogeneity among experiences of violence [1]. The concept of violence *severity* has been operationalized across studies differently, including by higher frequencies, more serious injuries, potential to cause harm, presence of a weapon, or accounts of the target or perpetrator only [19]. Some scales have pre-classified specific acts as more or less severe based on independent assessment of potential to cause harm, or egregiousness, by persons who may never have had similar experiences [20–22]. When measures of violence severity do not consider the perspective of the target or perpetrator, the classification of severity is arbitrary because it is not made relative to an individual’s context [6]. Severity is a complex construct with potential to reflect a continuum in problem magnitude through multiple dimensions including frequency and multiplicity of behaviors, subjective appraisal of the problem seriousness, polyvictimization, and intrusiveness of behavior [5].

#### Summary

Together, these conceptual underpinnings informed the dimensions of CLVS included in our scale development. We also drew on our 20-year history in studying violence and health for women and men, using qualitative and quantitative methods [23–25]. The scale development process included multiple steps, each of which was approved by our institution’s research ethics board. The conceptualization evolved and was revised at each step resulting in the final scale evaluated here. This work was conducted as part of the *Men’s Violence, Gender and Health Study (MVGHS)*, conducted in Eastern Canada with 685 men ages 19 to 65.

## Methods

### Development and pretesting

We defined cumulative lifetime violence as men’s *perceived* physical, psychological, and sexual violence experiences from childhood (under 18 years) through adulthood, as target and/or perpetrator, and in the context of gender, families, intimate relationships, schools, communities, and workplaces We developed items reflecting the dimensions of this conceptualization and had them vetted by local experts including individuals who identified as men, with attention to language that was meaningful for English-speaking men. We grouped items by salient dimensions of severity; that is, lifespan category (childhood, adulthood), focus (target, perpetrator) and type (physical, psychological, sexual). Each item also reflected a specific violence context (e.g., family, peer, intimate partner, workplace, community). We measured these dimensions on 5-point Likert-like scales by asking men to indicate their perceptions for each item of *how often* (never to very often) it occurred and *how distressing* (not at all to very) it was. Altogether, the nature of the items and their related measurement scales capture our conceptualization of violence severity (See Table 1). We were also mindful of potential respondent burden from including too many items.

**Table 1:** Dimensions of Cumulative Lifetime Violence Severity

	CHILD TARGET Frequency & Distress	CHILD PERPETRATOR Frequency & Distress	ADULT TARGET Frequency & Distress	ADULT PERPETRATOR Frequency & Distress
PHYSICAL VIOLENCE (includes violent acts or threats)	From someone older with power over me		As part of team or group, from someone	As team or group member, towards someone
	As part of team or group, from a peer	As team or group member, toward someone	From date or partner	Toward a date or partner
	From a date	Toward a date	From a boss or co-worker	Toward someone at work
	At school, home or community from a peer (not dating partner or team/group)	At school, home or community toward a peer (not dating partner or team/group)	As part of the nature of his work (e.g., military, policing, health care)	Used physical violence to control situation as part of his work
	Was harassed or stalked	Harassed or stalked another person	In public places (e.g., street, bars, sporting events, concerts)	Toward someone at home or in public places (not team/group or dating)
			From someone, during unrest (e.g., civil and political conflicts, jail, war)	Towards someone in situations of unrest such as civil and political conflicts, jail, or war
			Was harassed or stalked	Harassed or stalked someone
			From a caregiver or family member (not partner)	
PSYCHOLOGICAL VIOLENCE (includes yelled at, put down picked on, isolated, taunted, made to feel afraid, uncomfortable or controlled, overly criticized)	From someone older with power over me		From someone at work based on gender, sexual orientation, or other quality	Toward someone at work based on gender, sexual orientation, or other quality
	Saw violence among family or those I lived with		From someone at work	Toward someone at work
	Saw violence in my community		As part of a team/group	As part of team/group, toward someone
	As part of a team/group, from peers	As part of a team/group toward someone	Received messages or photos meant to hurt, control, put down	Sent messages or photos meant to hurt, control, put down
	Received messages or photos meant to hurt, control, put down	Sent messages or photos meant to hurt, control, put down	From a date or partner	Toward a date or partner
	From a date	Toward a date	From someone other than from dating/partner, at work or within team/group.	Toward someone other than a dating/partner, at work or within team/group
	At school, home or community from a peer (not dating partner or team/group)	At school, home or community toward someone (not dating partner or team/group)	Saw violence in my community	
			Saw violence among family or those I lived with	
SEXUAL VIOLENCE (touch against will in sexual way or pressure (threats, force, drugs/alcohol) into sexual activity)	From someone older with power over		As part of a team/group	As part of a team/group toward someone
	As part of team or group, from a peer	As part of a team/group toward someone	From a date or partner	Toward a date or partner
	From a date	Toward a date	From someone at work	Toward someone at work
	At school, home or community from a peer (not dating partner or team/group)	At school, home or community toward someone (not dating partner or team/group)	From someone other than from dating/partner, at work or within team/group.	Toward someone other than a dating/partner, at work or within team/group.

#### Pretest

Revisions based on feedback from experts resulted in a pilot measure of 49 items divided into 4 subscales: childhood target (13 items); adult target (16 items); childhood perpetrator (8 items); adult perpetrator (12 items). We pretested the measure with a convenience sample of 53 English-speaking men, ages 19 to 65 years, who resided in New Brunswick (NB) and agreed to participate in a pilot study of gender, violence, and health. Acceptable levels of internal consistency (Cronbach’s alpha = > .70) were obtained for all frequency and distress scales except childhood perpetration frequency (α = .63). Concurrent validity was supported by statistically significant positive correlations among the violence subscales and measures of depression and pain, suggesting men reporting more violence, whether as a target or perpetrator, tended to report higher levels of depression and pain. Lack of variability in men’s responses raised questions about the utility of some items and resulted in modification and/or deletion. The greatest variability in responses and highest scores were observed for the childhood violence target subscale, perhaps suggesting men were more willing to report childhood target experiences.

Through examination and discussion, we recognized that in an effort to reduce burden for respondents, we had included some items that tapped into multiple types of violence (i.e., physical, psychological, sexual) in more than one context. We reworked items so that each was conceptually distinct and represented only one cell in the CLVS dimension table (see Table 1). Specifically, we refined each item to measure one type of violence, in childhood or adulthood, as target or perpetrator, in one particular context (e.g., family, peer, workplace) to be scored in terms of *frequency* and *distress*. By averaging the *frequency* and *distress* scores for each item, we created a score for perceived violence *severity*; that is, how harsh, serious, forceful, unpleasant, and/or demanding that violence experience was felt to be by the respondent. This takes into account that distress initiates negative biophysical responses and permits the capture of the heterogeneity of men’s experiences that is often overlooked by using frequency only. For example, a man who scored *frequency* as rarely (2), and *distress* as very often (5) would have a *severity* score of 3.5, whereas another man who scored *frequency* as rarely (2), and *distress* as rarely (2 would have a *severity* score of 2. The subsequent revised CLVS scale had 64 items.

#### Test-retest

To explore stability of the measure, we recruited 31 additional NB men to complete CLVS-64 scale twice, 2 weeks apart. The test-retest reliability of the perceived CLVS-64 scale was *r*_s_ = .88, suggesting stability in the measure. Variability in responses remained poor for some items measuring sexual violence and for others measuring perpetration, with scores tending to be skewed toward the low end of the scale. Conceptually, these items were vital for capturing all dimensions of cumulative lifetime violence. We concluded that the poor distribution might be a result of the small sample, and opted to retain these items for the main study.

### The Main study

The *MVGHS* was conducted between April 2016 and March 2018 with a convenience community sample of individuals who identified as men, and were English-speaking residents of Eastern Canada, between the ages of 19 and 65 years willing to take part in a study of gender, health, and violence. Because we perceived violence to be pervasive in men’s lives, were interested in the relationship between severity of violence and health, and wanted a sample with wide variability in violence severity exposure, we did not use self-identifying a history of violence as an inclusion criterion. For scale development, our goal was to recruit a sample of 600, large enough to use principal components analysis (PCA) to reduce the number of scale items, while retaining conceptually important dimensions of CLVS-64 (See Table 1).

#### Measures

The study survey included the CLVS-64 as well as self-report questions on demographics, health, and gender. For each CLVS-64 item, we asked men to score on a 5-point Likert-like scale how often *(never* to *very often*) and how distressing (*not at all* to *very*). Because there is no existing measure of cumulative lifetime violence severity reflecting a gold standard for criterion validity, we used 4 global measures, each using a 10-point rating scale, for the frequency and distress of lifetime experiences of violence as perpetrator and target to determine convergent validity for the scale [26]. We summed and averaged these global measures for a Global Lifetime Violence Severity (GLVS) score of 0 to 10. We assessed concurrent validity with measures of depression, anxiety, and posttraumatic stress disorder (PTSD) because the relationship between each of these mental health problems and men’s exposure to violence such as IPV [27], workplace bullying [23] and child maltreatment [28] is well-established.

The Center for Epidemiological Studies Depression Scale Revised (CESD-R) is a 20-item 4-point Likert-type scale (*rarely* to *most of the time*) to assess depressive symptom frequency in past 2 weeks [29]. Summative scores range from 0 to 60 with higher scores indicating more depressive symptoms. Van Dam and Earleywine reported reliability and validity among diverse gender, age and community samples [30]. In this study, α = .95 (*N* = 685) [30]. The Generalized Anxiety Disorder Scale (GAD-7) is a 7-item, 4-point Likert-type scale (*not at all* to *nearly every day*) to measure severity of anxiety symptoms over the previous 2 weeks following DSM-IV criteria [31]. Summative scores range from 0 to 21, with higher scores indicating greater severity of symptoms. Reliability and construct validity have been established in the general population [32]. In this study, α = .94 (*N* = 685). The Posttraumatic Stress Disorder Checklist, Civilian Version (PCL-C) is a 17-item, 5-point Likert-type scale (*not at all* to *extremely*) to measuring how much the respondent has been bothered by PTSD symptoms based on DSM-IV criteria over the past month [33]. Summative scores range from 17 to 85 with higher scores reflective of greater symptomology [34]. Conybeare et al. reported good internal consistency, test-retest reliability, and convergent and discriminant validity in a non-clinical sample [35]. In the current study, α = .95 (*N* = 685).

#### Recruitment, data collection and data preparation

We recruited and collected data in two steps. First, we recruited men from the province of NB using posters and online classified advertisements. Using phone or email, interested men contacted the research coordinator who forwarded a letter of information and an online link for eligibility and consent. After receiving their consent, the research coordinator directed participants to the online survey. Of 825 men who were eligible and gave informed consent, 611 (74%) completed the survey and received an honorarium of 20 Canadian dollars to acknowledge their time.

Our missing data were minimal (i.e., ≤ 5%). Missing values were replaced by case mean substitution if case missing values were fewer than 30% in validated health scales and 20% in survey-specific violence scale items [26]. The sample size with complete data on violence items was 590, large enough to develop a stable factor analysis solution [36]. Corrected item-scale correlations are an indicator of how well the item measures the construct; experts differ on the cut score of corrected item-scale correlations for deletion of items with some indicating .20 and others .30 [26]. For the CLVS scale, only 11 items had scores less than .30, and 5 of these, all focusing on sexual violence, had scores less than .20. Conceptually, retention of sexual violence items was important for this scale; therefore, deletion of these items was not feasible. The mean inter-correlation among the 64 items was low at .195 with a range of .06 to .768. Although a number of correlations were less than .30 suggesting some items might have limited congruence with the scale, there was no evidence of over-redundancy as no items were correlated above .80 [26].

Examination of individual violence items revealed poor variability with distribution skewed toward the floor of the scale, particularly for some items focusing on sexual violence and/or on violence perpetration. Combining the upper scores of *often* and *very often* for frequency and *quite a bit* and *very* for how distressing resulted in improvement in distribution; however, sexual violence items and some perpetration items still failed to use the highest possible score and were skewed toward the lowest scale score. Normal distribution of variables is not necessary if PCA is used to summarize the relationships among a large set of observed variables but can enhance the solution [36]. However, Field noted that if the intent is to use the analysis beyond the sample collected, roughly normally distributed data is more important [37]. Since this analysis is the first step toward developing a measure of CLVS to be widely used with men, better distribution of data was judged to be important. Therefore, we recruited another 100 participants to complete the online survey. We modified the inclusion criteria to include participants living in the provinces of Nova Scotia and Prince Edward Island and required self-identification of having experienced violence in their lifetime. We reasoned that a broader catchment area would increase recruitment and that those who self-identified lifetime violence experience might be more likely to have responses toward the higher end of the scale. After data cleaning, the total sample size was 685. For a descriptive profile of the participants in the study, see Table 2. Of the 685 men, only 15 (2.2%) reported no experiences of violence in their lifetimes; 567 (82.8%) reported experiences as target and as perpetrator, 100 (14.6%) as target only, and 3 (0.4%) as perpetrator only.

**Table 2:** Descriptive Profile of Participants (*N* = 685)^a^

Age in Years: μ (range)	37.57 (19 to 65)
Cultural Affiliation: *n* (%)	(*n* = 684)
Anglophone	565 (82.5)
Francophone	52 (7.6)
First Nations	16 (2.3)
None of the above	51 (7.4)
^b^ Sexual Identity: *n* (%)	(*n* = 682)
Straight	612 (89.3)
Gay	38 (5.5)
Bisexual	27 (3.9)
None of the above	12 (1.8)
Marital Status: *n* (%)	(*n* = 682)
Single, never married	207 (30.2)
Married	242 (35.3)
Living with partner	157 (22.9)
Separated	35 (5.1)
Divorced	41 (6.0)
Dependents (under 18 years): *n* (%)	(*n* = 680)
Yes	242 (35.3)
No	242 (63.9)
Community Size: *n* (%)	(*n* = 684)
Rural (less than 1000)	88 (12.8)
Small town (1000 to 29,999)	152 (22.2)
Medium City (30,000 to 99,999)	358 (52.3)
Large City (100,000 or more)	86 (12.6)
Highest Level of Education: *n* (%)	(*n* = 684)
Less than high school diploma	53 (7.7)
High school diploma	116 (17.0)
Some post-secondary education	195 (28.5)
College diploma or university degree(s)	320 (46.7)
Currently Employed: *n* (%)	
Yes	472 (68.9)
No	213 (31.1)
^c^ Total Personal Income in Past Year: *n* (%)	(*n* = 678)
Less than $10,000	124 (18.1)
$10,000 to $24,999	167 (24.4)
$25,000 to $49,999	161 (23.5)
$50,000 to $74,999	126 (18.4)
$75,000 to $100,000	53 (7.7)
More than $100,000	47 (6.9)

^a^Unless otherwise indicated. ^b^ Categories are not mutually exclusive ^c^ Canadian dollar

In order to minimize skewness in the violence items, we recoded violence *frequency* and *distress* scores to a 4-point scale in which 4 represented the upper two scores of the original scale and calculated *severity* scores (range 1 to 4) for each item by averaging the *frequency* plus *distress* score. With respect to normality in item distribution in the sample of 685, 58 of 64 items used the full range of *severity* scores from 1 to 4, with 5 items having a maximum score of 3.5, and 1 having a maximum score of 3. Although Kline suggested that a skewness score greater than 3 is an indicator for deletion of items, we retained the 14 items that exceeded 3 because most were about sexual violence and considered conceptually important [38]. Of the 64 items, 7 had corrected item-scale correlations less than .3 and all measured sexual violence. One, perpetration of sexual violence at work, had a corrected item-scale correlation of less than .2 and was deleted from the scale because it was unlikely to correlate with the other items [26, 36]. Among the remaining 63 items, the mean inter-item correlation was .218 (range − .023 to .754) and most were significant (<.05). Significance suggested reliability in relationships among pairs of items, an indicator of the matrix being factorable [36]. As well, a matrix should include several sizable (>.30) inter-item correlations, although larger sample sizes may produce smaller correlations. In our sample, 57 items had correlations above .30 with at least 4 and as many as 26 other items suggesting that the items overall are suitable for factor analysis. Thus, data were deemed suitable for PCA.

#### Principal components analysis

Using an oblique rotation to begin, we found correlations among components to be, with one exception, less than .30 indicating the components were largely uncorrelated; therefore, we judged a PCA with orthogonal rotation to be most appropriate [39]. With 63 items, we ran the PCA with orthogonal varimax rotation, specifying generation of a Scree plot, extraction of components based on eigenvalue > 1, display of coefficients sorted by size, and suppression of coefficients < .32 because these cannot not be interpreted in PCA [36]. This initial analysis provided further support for factorability of the data set. In the anti-image correlation matrix, the negatives of pairwise partial correlations coefficients adjusted for the effects of all other items were very small, indicating factorability. Additionally, the Kaiser-Meyer-Olkin (KMO) test, a ratio of the sum of squared correlations among items to the sum of squared correlations plus the sum of squared partial correlations, was .91; the closer this is to 1, the better for factor analysis [36, 39]. Additionally Measures of Sampling Adequacy (MSA), which is the KMO for individual pairs of items, were all above .810, greater than the .5 required for sampling adequacy [37]. The output from the initial Varimax rotation showed 14 components, all with eigenvalues > 1, that accounted for 62.85% of the variance. The Scree test showed a break in trajectory at 11 and 13 components. Thurstone as cited in Tabachnick and Fidell [36] suggested simple structure in the solutions; that is, each component should have several variables with high loadings (i.e., above .50) but each variable should have a primary or high loading on only one component. Following rotation, items without primary loadings on a single factor can be eliminated. Cross loadings (loadings on more than one component) that are separated by less than .20 from the primary loading are also problematic as these indicate a complex variable that contributes to more than one construct. We aimed for a simple solution by deleting items one at a time, first on the basis of lacking a primary loading greater than .50, and then on the basis of cross loadings separated by less than .2 from the primary loading. We ran a new PCA after each item was deleted. We deleted items starting from the bottom of the rotated factor matrix, reasoning that the components at the bottom accounted for less variance, consisted of fewer items, and likely were less stable.

Using this approach, we ran many PCAs with the 63 variables using varimax and quartimax rotations and examined solutions with differing numbers of components. Varimax rotations provided simple solutions that were quite consistent across 9, 10, and 11 factor components, although we judged the 11component, 44-item solution to be the best match for our conceptualization of CLV. To confirm this solution, we also randomly selected 400 cases from the sample of 685 and ran the PCA first on the 400 and then on the remaining 285. The 400-case PCA yielded a 10 component, 35-item solution and the 285-case PCA yielded a 9 component, 33 item solution. Both were remarkably similar to the 11 component solution with components that accounted for the majority of the variance similar in all three solutions. The consistency across solutions even with the smaller samples provided support for the original 11 component solution.

## Results

Within the 11 component, 44-item (CLVS-44) solution, each item loaded primarily on one component and none had cross loadings with less than .20 separation from the primary loading (See Table 3). Sampling adequacy was very good as indicated by the KMO of .87 and MSAs greater than .76. All components had eigenvalues greater than Kaiser’s criterion of 1, and together explained 64.06% of the variance. Variance explained by individual components can be found in Table 3. The fit of this model was good; only 15% of the non-redundant residuals between the observed and reproduced correlations were greater than .05 [37].

**Table 3:** Varimax Rotated Component Matrix—CLVS-44 (11 Component, 44 Item)

Scale Items	% of Variance	α	Rotated Component Matrix										
			1	2	3	4	5	6	7	8	9	10	11
Lifetime Perpetrator Physical & Psychological Violence (not partner or work)	**8.36**	**.85**											
Before the age of 18, at school, home or in the community (other than in a dating relationship or within a team/group), I physically threatened or was physically violent toward someone.			.740										
Since the age of 18, other than in a dating or partner relationship, at work or as part of a team/group, I have physically threatened or physically hurt someone at home, or in public places, such as on the street, in bars, at sporting events or concerts.			.712										
Before the age of 18, at school, home or in the (other than in a dating relationship or within a team/group), I yelled at, taunted, isolated, left out, put down, picked on, controlled or threatened someone.			.700										
Before the age of 18, as a part of a team or group, I physically threatened or physically hurt someone in a way that ‘crossed the line’.			.638										
Since the age of 18, other than in a dating or partner relationship, at work or as part of a team/group, I have yelled at, taunted, isolated, put down, picked on, controlled or threatened someone.			.632										
Since the age of 18, as part of a team or group, I have physically threatened or physically hurt someone in a way that ‘crossed the line’.			.575										
Childhood Target Physical & Psychological Peer/Team Violence	**6.98**	**.86**											
Before the age of 18, as a part of a team or group, I was the target of criticism or comments from another child/peer that felt uncomfortable or that ‘crossed the line.’				.780									
Before the age of 18, as part of a TEAM or GROUP, I was physically threatened or physically hurt by another child/peer in a way that ‘crossed the line.’				.766									
Before the age of 18, at school, home or in the community (other than in a dating relationship or within a team/group), I was yelled at taunted, isolated, put down, picked on, or scared by another child/peer.				.765									
Before the age of 18, at school, home or in the community (other than in a dating relationship or within a team/group), I was hit, kicked, slapped, burned, choked or physically hurt by another child/peer.				.743									
Lifetime Perpetrator Sexual Violence	**6.73**	**.80**											
Since the age of 18, as a part of a team or group, I have touched someone against their will in a sexual way or forced/pressured someone into sexual activity by using threats, physical force, pressure or drugs/alcohol.					.773								
Before the age of 18, as a part of a team or group, I touched someone against their will in a sexual way or forced/pressured someone into sexual activity by using threats, physical force, pressure or drugs/alcohol.					.766								
Before the age of 18, at school, home or in the community (other than in a dating relationship or within a team/group), I made someone take part in sexual activity or have sex against their will by using threats, physical force, pressure or drugs/alcohol.					.749								
Since the age of 18, other than in a dating or partner relationship, at work or as part of a team/group, I have touched someone against their will in a sexual way or forced/pressured someone into sexual activity by using threats, physical force, pressure or drugs/alcohol.					.656								
Since the age of 18, in a dating or partner relationship, I touched someone against their will in a sexual way or forced/pressured someone into sexual activity by using threats, physical force, pressure or drugs/alcohol.					.607								.331
Adult Target Psychological Violence Work, Messaging, Stalking	**6.18**	**.78**											
Since the age of 18, at work I have been put down, overly criticized, controlled, isolated, or made to feel small						.661							
Since the age of 18, at work I have been taunted, called names or treated meanly, based on my gender, sexual orientation, or other qualities.						.637							
Since the age of 18, I have been the target of messages or photos that were meant to hurt, scare, control, or put me down (such as written notes, texts, or social media).						.634							
Since the age of 18, I have been harassed or stalked.						.604							
Since the age of 18, other than in dating or partner relationships, at work or within teams/groups, I have been yelled at, put down, isolated, controlled, or made to feel afraid.						.568							
Childhood Target Sexual Violence	**6.15**	**.80**											
Before the age of 18, I was touched against my will in a sexual way or forced or pressured into sexual activity by someone with power over me (such as, parent, caregiver, teacher, coach, or someone older).							.809						
Before the age of 18, at school, home, or in the community (other than in a dating relationship or within a team/group), I was touched against my will in a sexual way or forced or pressured into sexual activity by another child/peer.							.720						
Before the age of 18, as a part of a team or group, I criticized, or made comments that made someone feel uncomfortable or that ‘crossed the line’.			.381				.676						
Before the age of 18, As part of a team or group, I was touched against my will in a sexual way or forced/pressured into sexual activity by another child/peer.							.667						
Adult Target and Perpetrator Violence related to Nature of Work or Civil/Political Unrest	**5.93**	**.81**											
Since the age of 18, my job (for example, military, police, health care) has required me to use physical violence to control a situation								.811					
Since the age of 18, I have experienced physical violence due to the nature of my work, for example, military, policing, health care.								.804					
Since the age of 18, I have been physically threatened or experienced physical violence in situations of unrest, such as civil and political conflicts (strikes, protests), jail, or war.								.703					
Since the age of 18, I have physically threatened or been physically violent toward someone in situations of unrest, such as civil and political conflicts (strikes, protests), jail or war.			.364					.654					
Lifetime Target Physical and Psychological Dating/Partner Violence	**5.73**	**.78**											
Before the age of 18, I was hit, kicked, slapped, burned, choked or otherwise physically hurt by someone I dated.									.760				
Before the age of 18, I was yelled at, put down, isolated, made to feel afraid or controlled by someone I dated.									.721				
Since the age of 18, in a dating or partner relationship, I have been hit, kicked, slapped, burned, choked or otherwise physically hurt.									.675				
Since the age of 18, in a dating or partner relationship, I have been yelled at, put down, isolated, controlled, or made to feel afraid.						.375			.655				
Lifetime Target Family Physical Violence	**5.44**	**.78**											
Before the age of 18, I saw violence (such as bullying, threats, physical or sexual assault, or harassment) among my family members, or those I lived with.										.723			
Since the age of 18, I have seen violence (such as bullying, threats, physical or sexual assault, or harassment) among my family members, or those I lived with.										.651			
Since the age of 18, I have been hit, kicked, slapped, burned, choked or otherwise physically hurt by a caregiver or family member (other than a partner).										.634			
Before the age of 18, I was hit, kicked, slapped, burned, choked or otherwise physically hurt by someone with power over me (such as, parent, caregiver, teacher, coach, or someone older).				.404						.607			
Lifetime Perpetrator Stalking and Messaging	**5.21**	**.72**											
Since the age of 18, I have sent written notes, texts or messages or photos by social media to hurt, put down, scare or control someone.											.722		
Since the age of 18, I have harassed or stalked another person											.693		
Before the age of 18, I harassed or stalked another person.											.625		
Before the age of 18, I sent written notes, texts, or messages or photos by social media to hurt, put down, scare, or control another person.											.568		
Adult Perpetrator Psychological Workplace Violence	**3.70**	**.69**											
Since the age of 18, at work I have taunted, called names, or treated someone meanly based on their gender, sexual orientation, or other qualities.												.750	
Since the age of 18, at work I have put down, overly criticized, controlled, isolated, or made someone feel small.												.717	
Lifetime Perpetrator Physical Dating/Partner Violence	**3.68**	**.69**											
Since the age of 18, In a dating or partner relationship, I have hit, kicked, slapped, burned, choked or otherwise physically hurt my partner.													.815
Before the age of 18, I hit, kicked, slapped, burned, choked or otherwise physically hurt someone I dated.													.657

Cronbach’s Alpha for the CLVS-44 was .92. The mean inter-item correlation was .214 and 4 sexual violence items had corrected item-total correlations of less than .3, although all were above .20. Cronbach’s Alpha would not have improved with the removal of any item. The Cronbach’s Alpha for each of 11 components can be seen in Table 3. Most corrected item-scale correlations were greater than .50 and no Cronbach’s Alpha had the potential to be strengthened by removal of an item. Notably, we found each component to be theoretically meaningful and applied a suitable label to each (See Table 3). Components 10 and 11 each had only 2 items, with alphas and mean inter-item correlations of .69 and .52 and .69 and .53 respectively. Although two-item components are not ideal because they may lack stability, and the alpha for each is slightly less than .70, we retained the components because they are conceptually important and the items are moderately correlated. The CLVS-44 included items for physical, psychological, and sexual violence, as a child and adult, and as a target and a perpetrator; however, one major gap was the lack of items retained for adult target of sexual violence.

For the 685 men, the mean score for the CLVS-44 was 1.35 (S.D. = .36; range = 1.00 to 2.97) and the GLVS Score had a mean of 3.23 (S.D. = .2.34; range = 0 to 10). The CLVS-44 was supported by convergent validity between the two scales of *r =* .750 (*p* < .001)*.* The health measures had the following total mean scores: CESD-R (μ =15.33, S.D. = 14.41; range = 0.00 to 60.00), PCL-C (μ =34.07, S.D. = 15.25; range = 17.00 to 83.00), GAD-7 (μ =5.79, S.D. = 5.81; range = .00 to 21.00). Concurrent validity of the CLVS-44 with the CESD-R was *r =* .482 (*p* < .001), with the PCL-C was *r =* .592 (*p* < .001), and with the GAD-7 was *r =* .477 (*p* < .001). These moderate correlations with mental health problems known to be associated with experiences of violence provide support for concurrent validity of this new measure. Finally, concurrent and convergent validity for each component was examined with the same measures and all were significant (See Table 4).

**Table 4:** Construct and Concurrent Validity for 11 Components of the CLVS-44

Components	CESD_R	PCL-C	GAD-7	GLVS
Lifetime Perpetrator Physical & Psychological Violence (not partner or work)	.258^a^	.340^a^	.292^a^	.530^a^
Childhood Target Physical & Psychological Peer/Team Violence	.338^a^	.416^a^	.350^a^	.623^a^
Lifetime Perpetrator Sexual Violence	.119^a^	.143^a^	.089^b^	.171^a^
Adult Target Psychological Violence Work, Messaging, Stalking	.405^a^	.486^a^	.410^a^	.562^a^
Childhood Target Sexual Violence	.288^a^	.377^a^	.273^a^	.396^a^
Adult Target and Perpetrator Violence related to Nature of Work or Civil/Political Unrest	.170^a^	.263^a^	.193^a^	.376^a^
Lifetime Target Physical and Psychological Dating/Partner Violence	.391^a^	.426^a^	.356^a^	.447^a^
Lifetime Target Physical Violence	.388^a^	.488^a^	.404^a^	.603^a^
Lifetime Perpetrator Stalking and Messaging	.312^a^	.337^a^	.251^a^	.358^a^
Adult Perpetrator Psychological Workplace Violence	.198^a^	.249^a^	.164^a^	.366^a^
Lifetime Perpetrator Physical Dating/Partner Violence	.186^a^	.240^a^	.180^a^	.324^a^

^a^Significant at 0.01 (2-tailed)

^b^Significant at 0.05 (2-tailed)

## Discussion

The CLVS-44 is, to our knowledge, the first measure of cumulative lifetime severity for men where the total score reflects a comprehensive model of lifetime violence severity including dimensions of type, focus (perpetrator or target), timing (childhood or adulthood), context, frequency, and degree of distress. Total CLVS-44 scores offer a way of examining relationships between cumulative lifetime violence severity and health that takes into account multiple diverse, concurrent, and recurrent experiences of violence and men’s perceptions of them. This new measure may help to overcome the current problem of attributing health problems to one or two violence exposures at particular points in the life course while ignoring the intersecting and augmenting effects of other violence experiences. Unlike most other measures of violence, the CLVS-44 includes experiences as target and perpetrator, thus broadening potential for understanding how both contribute to health. Our finding that 82.8% of men in the study reported experiences as both target and perpetrator supports the importance of including both in this measure.

Additionally, the nature of the items in each of the 11 components reveals interesting patterns of violence severity. Several components capture lifetime patterns of violence severity; that is, experiences that are common both in childhood and adulthood such as Lifetime Perpetration of Physical and Psychological Violence, Lifetime Perpetration of Sexual Violence, and Lifetime Target of Family Physical Violence. Three components include both physical and psychological violence types; for example, Childhood Target Physical and Psychological Peer/Team Violence and Lifetime Perpetration Physical and Psychological Dating and/or Partner Violence. Others focus strictly on one type of violence at one point in the lifespan such as Childhood Target Sexual Violence and Adult Perpetrator Psychological Workplace Violence. Only one component combined items that captured both target and perpetration, Adult Target and Perpetrator Violence related to Nature of Work or Civil/Political Unrest. Examination of each component offers potential insights regarding similarity among items. It is possible that the patterns of cumulative lifetime violence indicated by particular components or groups of components may have implications for health outcomes that facilitate interpretation of the total CLVS-44 scores. Thus the present PCA analysis adds to knowledge by permitting not only the calculation of a total CLVS-44 score but also of separate component scores that may reveal unique patterns of violence associated with particular health outcomes.

### Limitations and future research

This measure does not differentiate between males and females as perpetrators or targets, a shortcoming that may reduce its usefulness for some purposes. A significant limitation of our analysis is that no items reflecting adult experiences as target of sexual violence were retained in the PCA. This may be sample-specific; perhaps because having experienced violence was not an inclusion criterion for most of the sample, levels of violence, particularly being targeted as adults for sexual violence, were low for many participants. Reporting experiences of sexual violence is difficult for adult men and possibly hampered by societal perceptions that men do not have non-consensual sexual experiences [40]. At each stage in the development of this measure, we encountered methodological challenges such as lack of variability or low corrected inter-item correlations with items designed to capture sexual violence, but kept the items because sexual violence is a critical conceptual dimension. Despite our findings here, our future testing of this measure will include these adult sexual target items because they are conceptually important and because PCA has some limitations. Further ways to determine the utility of this tool for capturing adult target for sexual violence could be to recruit a sample of men who self-identify with a history of violence and/or a clinical sample of men seeking help for experiences of sexual violence.

PCA analysis is based on the assumption that the sample used is the population; because our sample is a community convenience sample, our conclusions are not generalizable beyond this sample [37]. Future research is needed for cross-validation with other diverse samples. By conducting initial testing of the CLVS measure with a community sample of 685 men ages 19 to 65 years, our results may be more robust than those that might have emerged from restricted samples such as college students or clinical samples commonly used in scale development. Nonetheless, our sample of Eastern Canadian men is unique; specific characteristics can be seen in Table 2. Most men in this study lived in rural areas, small towns and medium-sized cities, and not in large ethnically-diverse metropolitan areas where much research focusing on violence and health is conducted. Another difference is cultural context; NB is the only officially bilingual Canadian province. Most of the population is white and the dominant cultures are Anglophone and Francophone. Additionally, our sample ranged from age 19 to 65, and tended to be younger than Eastern Canadian men in general. Thus, although this community sample incorporates some unique diversity, findings from this study may be limited in their application, particularly to men living in metropolitan areas, who are older, and/or have other ethnic heritage. Still scale development is an incremental process that takes place through testing in multiple samples and we believe that this study is a strong contribution to the development of a CLVS scale.

Going forward with our program of research, we will examine the relationships between CLVS-44 scores and specific health outcomes such as chronic pain, cannabis use, cardiovascular risk, and depression and determine the utility of the CLVS-44 for multivariate studies examining potential mediators and moderators of these associations. Moreover, for those health outcomes that are significantly associated, we will also examine patterns of associations with CLVS-44 components or subscales. Identification of components that have the most influence on specific health outcomes may be particularly useful for developing clinical protocols for providing trauma and violence-informed care for men [41].

Next steps will be to test the measure including adult sexual target items in a large (*N* = 1400) sample of men ages 19 and older, living anywhere in Canada, who self-identify as having experiences of violence. Stratified or quota sampling based on social location such as age, community size, ethnic diversity, and income as well as experiences as target for adult sexual violence may be used to increase applicability of findings. The sample will be randomly divided into two groups, with a PCA run on one group to determine component structure and a confirmatory factor analysis of that theoretical structure on the second group. By including the sexual target items, we will determine whether this was an artifact of the particular sample, or is common across men.

## Conclusion

The CLVS-44 scale is a step forward in the study of violence as a social determinant of health because it offers a comprehensive total CLVS score amenable to multivariate analyses. No other measure of perceived violence severity that we could locate included items for type (physical, psychological, sexual), timing (childhood, adulthood), focus (target, perpetrator), and context (e.g., workplace, intimate relationship, family, community). Additionally, because the severity score for each item is based on perceived frequency and perceived distress, the total score captures the mechanism by which violence severity affects health. Future research with diverse samples of men is necessary to determine the utility of the CLVS-44 for explaining relationships between CLVS and specific health outcomes.

## Data Availability

The data set used and analysed during the current study is available from the corresponding author on reasonable request. The Cumulative Lifetime Violence Severity-44 scale is available from the corresponding author on request.

## Abbreviations

ACE: Adverse Childhood Events
CESD-R: Center for Epidemiological Studies Depression Scale Revised
CLVS: Cumulative lifetime violence severity
GAD-7: Generalized Anxiety Disorder Scale
GLVS: Global Lifetime Violence Severity Scale
IPV: Intimate partner violence
KMO: Kaiser-Meyer-Olkin test
MSA: Measures of Sampling Adequacy
MVGHS: Men’s Violence Gender and Health Study
NB: New Brunswick
PCA: Principal components analysis
PCL-C: Posttraumatic Stress Disorder Checklist, Civilian Version
PTSD: Posttraumatic stress disorder
